# Supplementary figures and images for: Functional CDKN2A assay identifies frequent deleterious alleles misclassified as variants of uncertain significance
Source: eLife. 2022 Jan 10;11:e71137. doi: 10.7554/eLife.71137 (PMC8824478; doi:10.7554/eLife.71137)

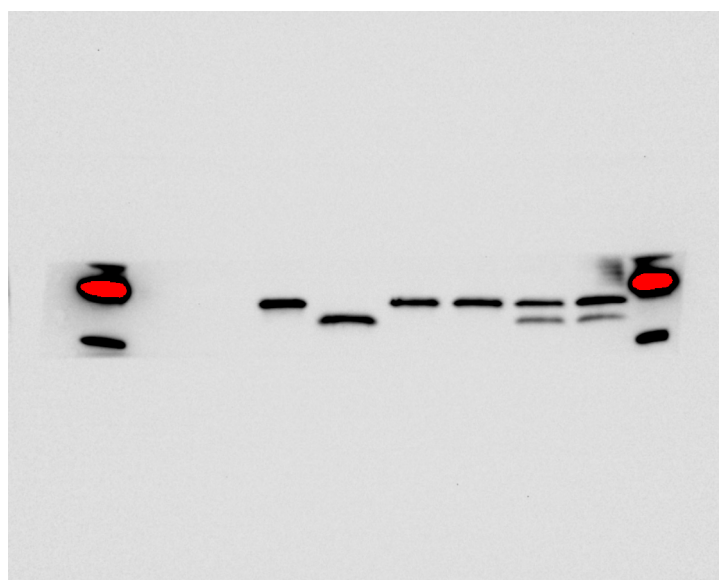

Supplement: Figure 2—source data 1. [file elife-71137-fig2-data1.zip › Figure 2source data 1.pdf]

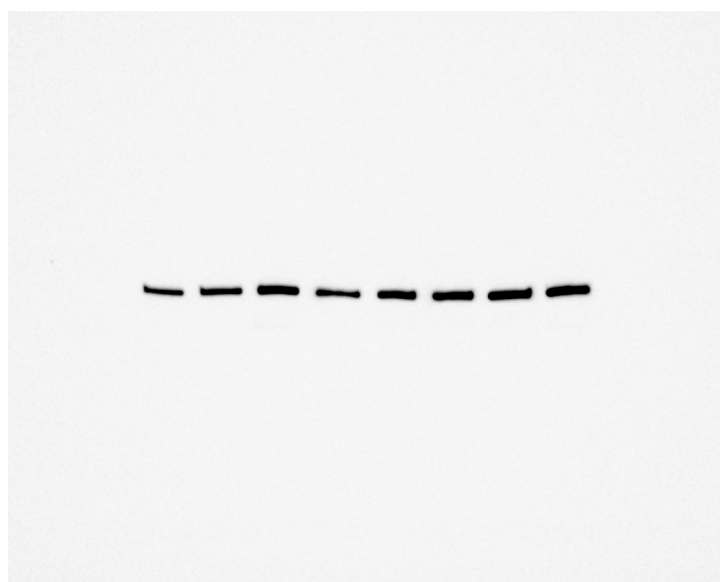

Supplement: Figure 2—source data 2. [file elife-71137-fig2-data2.zip › Figure 2source data 2.pdf]

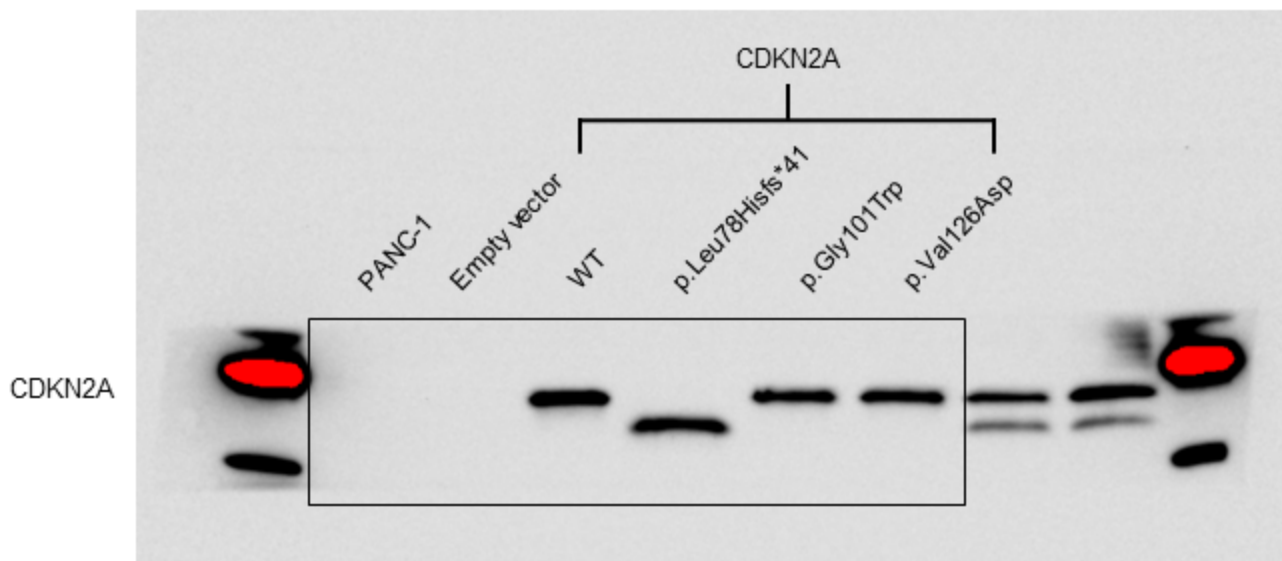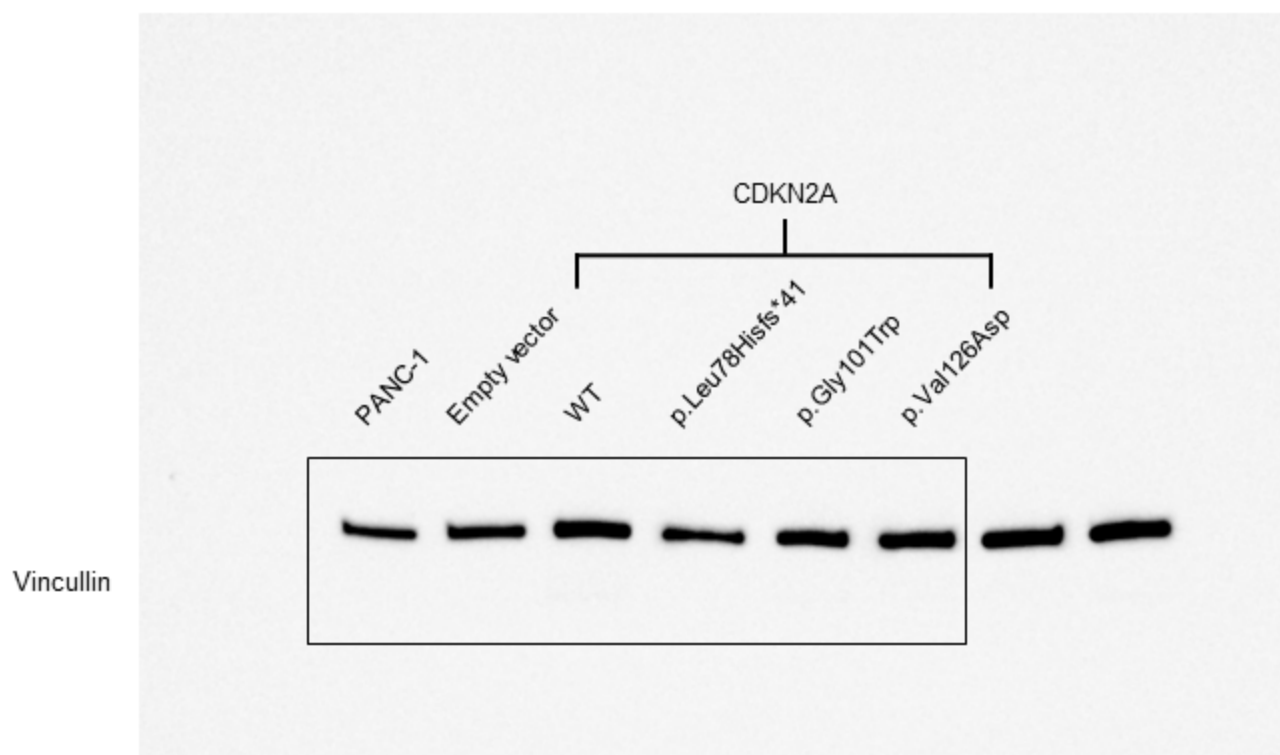

Supplement: Figure 2—source data 3. [file elife-71137-fig2-data3.zip › Figure 2source data 3.pdf]

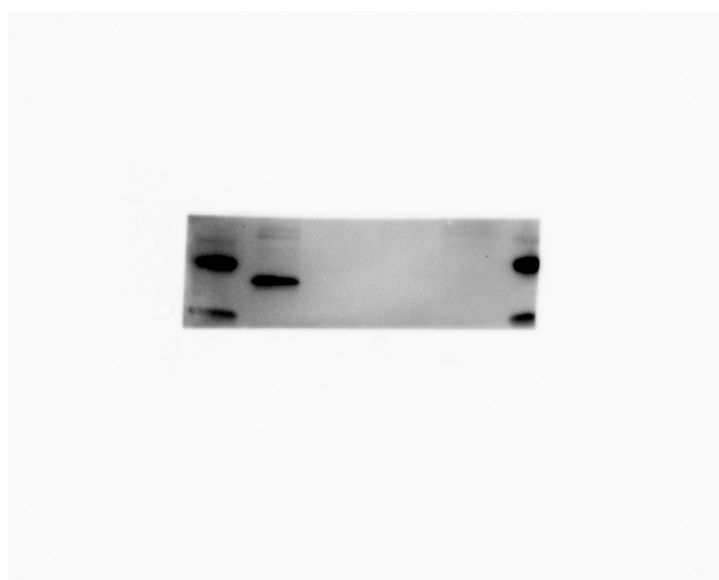

Supplement: Figure 3—figure supplement 1—source data 1. [file elife-71137-fig3-figsupp1-data1.zip › Figure 3figure supplement 1source data 1.pdf]

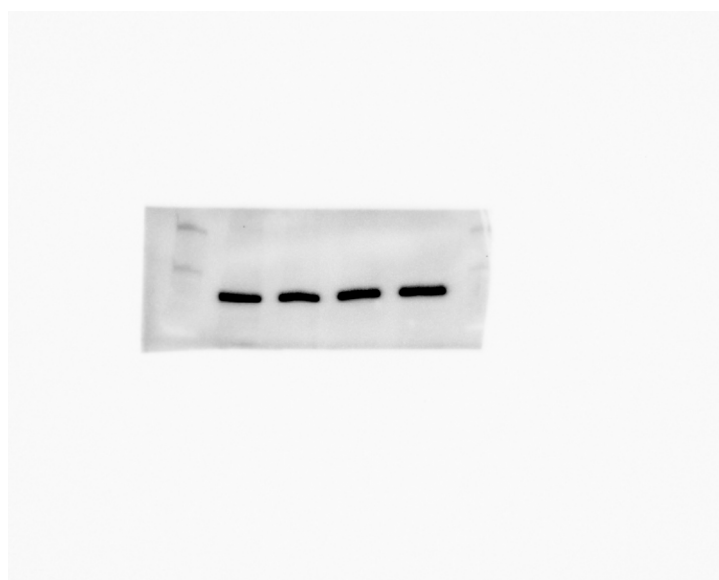

Supplement: Figure 3—figure supplement 1—source data 2. [file elife-71137-fig3-figsupp1-data2.zip › Figure 3figure supplement 1source data 2.pdf]

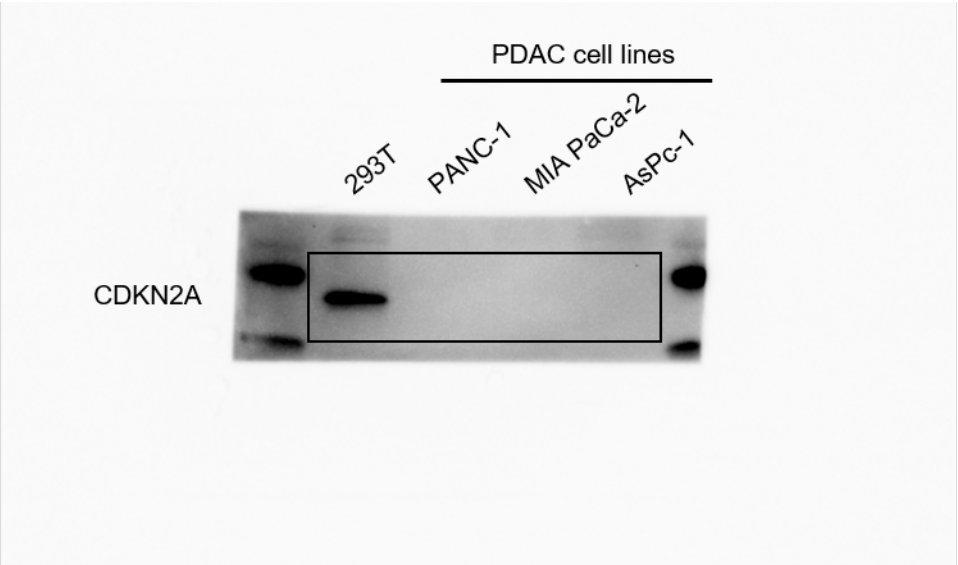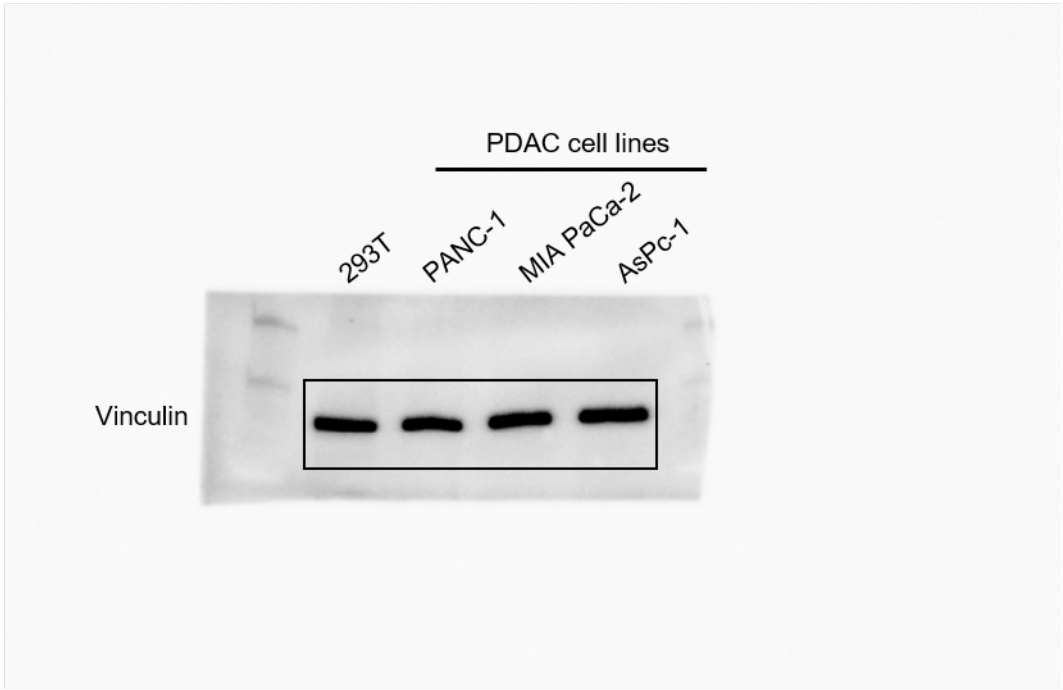

Supplement: Figure 3—figure supplement 1—source data 3. [file elife-71137-fig3-figsupp1-data3.zip › Figure 3figure supplement 1source data 3.pdf]
